# Microbiome and the future for food and nutrient security

**DOI:** 10.1111/1751-7915.12592

**Published:** 2017-01-11

**Authors:** Brajesh K. Singh, Pankaj Trivedi

**Affiliations:** ^1^ Hawkesbury Institute for the EnvironmentWestern Sydney UniversityPenrithNSWAustralia; ^2^ Global Centre for Land‐Based InnovationWestern Sydney UniversityPenrithNSWAustralia; ^3^ Department of Bioagricultural Sciences and Pest ManagementColorado State UniversityFort CollinsCOUSA

## Abstract

Microbiome and the future for food and nutrient security

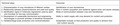

Global demands for food and fibre will increase up to 70% by 2050. This increase in agricultural productivity needs to be obtained from existing arable land, under harsher climate conditions and with declining soil and water quality. In addition, we have to safeguard our agricultural produce from new, emerging and endemic pests and pathogens. Harnessing natural resources including the ‘phytomicrobiome’ is proposed to be the most effective approach to improve farm productivity and food quality in a sustainable way, which can also promote positive environmental and social outcomes.

Conventional farming that uses chemicals in the form of fertilizers and pesticides has substantially increased agriculture productivity and contributed immensely to food access and poverty alleviation goals. However, excessive and indiscriminate use of these chemicals has resulted in food contamination, negative environmental outcomes and disease resistance which together have a significant impact on human health and food security. The microbiome technology has the potential to minimize this environmental footprint and at the same time sustainably increase the quality and quantity of farm produce with less resource‐based inputs.

Plants and associated microbiota evolved together and have developed a mutualistic relationship where both partners benefit from the association. However, plant breeding programmes have unintentionally broken this association, resulting in the loss of key beneficial members of the crop microbiome. From the limited knowledge obtained to date, it is evident that crop yields and fitness are linked to the plant microbiome. Harnessing the plant microbiome therefore can potentially revolutionize agriculture and food industries by (i) integrating crop health with better management practices for specific climatic conditions to improve productivity and quality; (ii) using environmental friendly approaches to control pests and pathogens and thus reduce the use of chemical pesticides with environmental and health implications; (iii) considering smarter and efficient methods for using natural resources including soil and water; (iv) producing a better quality of food with less chemical contamination and allergens; and (v) minimizing losses by improving crop fitness in extreme weather or future change scenarios.

## Rhizosphere versus phytomicrobiome approaches

The phytomicrobiome consists of microbiota associated with all plant compartments (e.g. root, stem, leaf, flower, seeds). However, the majority of research in this area is focussed on the rhizosphere microbiome, which drives key interface interactions between plant roots and soils in terms of resource acquisition and plant health. A body of work has demonstrated the key role of the rhizosphere microbiome in nutrient acquisition, disease resistance, resilience to abiotic stresses and fitness in novel environments. However, due to technical challenges the phytomicrobiomes of other plant‐associated niches (leaf, stem, endophytes) have received much less attention. Such bias is linked to technical challenges associated with characterizing leaf, stem and other parts of the plant. Amplifying bacterial marker genes (16S rDNA) from plant tissues is challenging as bacterial DNA is overwhelmed by the chloroplast and mitochondrial DNA that show high sequence similarities with Chlorobi/Chloroflexi/Cyanobacteria phyla. In recent years, the use of peptide nucleic acid (PNA) that blocks the amplification of contaminant sequences has helped to improve the efficiency of bacterial amplicon sequencing. The sequencing of fungal amplicons has been technically easier, the lack of universal primers to provide a consistent, unbiased overview limits the information on the fungal members of the phytomicrobiome. Application of technologies (such as shotgun sequencing) that can provide a comprehensive overview of the functional potential of the phytomicrobiome remains challenging given the microbiome sequences are masked by plant sequences resulting in extremely low coverage of the microbial metagenome from plant tissues. Technologies which can specifically enrich microbial DNA/RNA from plant materials are needed. Although with a low efficiency, some commercial kits selectively enrich bacterial mRNA and have the potential to circumvent this issue for the bacterial community to some extent; however, similar technologies are needed for the fungal phytomicrobiome given fungi play a significant role in both nutrient use efficiency and plant protection against biotic and abiotic stresses.

In addition to the technical issues highlighted, the lack of a holistic approach for plant microbiomes is based on the assumption that the rhizosphere microbiota plays the most important role in plant productivity. It can be argued that based on limited available evidence, the root (rhizosphere and root endophytes) may play a more important role in nutrient uptake, while other sections of the plant microbiome play a stronger role in the defence of pathogen and pest attacks, and resource use efficiency, thus affecting quality and quantity of plant yield. However, this may not be true in all cases and may be crop‐, region‐ and climate‐specific. Therefore, we firstly need a complete characterization of the phytomicrobiome associated with crop varieties and different compartments (e.g. leaf, stem, root) grown under different environmental and climatic conditions. This will allow us to characterize the core microbiome of crop species and distinguish between varieties and environmental conditions. Further characterization of the role of the core microbiota in crop fitness and yield, combined with identifying the metabolic pathways of the microbiome, will help in designing tools to manipulate the phytomicrobiome to sustainably increase agriculture productivity and the quality of food.

The uniqueness of the phytomicrobiome among different niches and varieties provides both opportunities and challenges for harnessing the phytomicrobiota for increasing agriculture productivity, improving quality of food and sustaining environmental functions. There is significant evidence to suggest that ecological functions performed by the phytomicrobiome extend a plant's ability to adapt to different environmental conditions and changes (Bulgarelli *et al*., 2013), which is of primary significance for the plant's fitness considering their sessile lifestyles. However, traditional crop breeding programmes do not consider key components of crop fitness, i.e. phytomicrobiota, and as a result, some weakness of crops to biotic and abiotic stress is attributed to this negligence. Excessive use of agrochemicals has also negatively impacted the strength of this relationship. Therefore, future breeding programmes will need to use a combination of genetic information from the host and metabolic pathways from the associated microbiomes. Such an approach is critical to ensure all intended benefits of breeding programmes without losing beneficial microbiota. This in turn potentially impacts plant fitness and resilience against biotic and abiotic stress. Going forward, the use of agrochemicals (particularly fertilizers) will remain an important ingredient for agriculture; however, its precision use combined with better chemistry and improved breeding programmes to explicitly consider the health of phytomicrobiome will be integral to sustainably increase agriculture productivity and food quality.

## Ability to manipulate the microbiome *in situ*

In recent years, our understanding has improved regarding the levels of soil biodiversity, drivers of biodiversity in agriculture systems and relationships between biodiversity and ecosystem functions including nutrient availability and agriculture productivity. Key knowledge on the critical role played by the microbial community in the rhizosphere, particularly in nutrient acquisition and disease resistance, has also improved. But our ability to manipulate the microbial diversity for improved production is either limited to altering management practices (i.e. tillage, residue retention, use of agrochemicals, etc.) or through the addition of microbial inoculates. The use of microbial inoculants has so far limited success in field conditions – mainly due to competition with the indigenous microflora of soils. However, there is strong evidence to suggest that plants and their associated microbiota (particularly of rhizosphere) constantly communicate with each other for resource requirements and defence against pathogen and parasite attacks. However, we have limited knowledge on the communication (signal) molecules used by plant or microbes for these communications. Identifying these signal molecules should be a primary focus of research as this can provide an effective tool for manipulating plant–microbe interactions for maximizing resource availability and plant protection. For example, signal molecules (or their inhibitors) could be used to specifically promote the activity of beneficial microbes, to increase microbial mobilization of nutrients (nitrogen and phosphorus) and to defend against pathogens and pests when needed (synchronized supply with demand). However, this is a significant challenge given that the quantity of signal molecules in root exudates and microbial biofilms is extremely low and is difficult to characterize by available technology. Along with increasing the sensitivity of different spectroscopies, an integrated approach of metagenomics, metatranscriptomics and metabolomics will be needed to characterize signal molecules, their diversity and specificity to harness these for improving farm yields and quality.

*In situ* microbiome engineering (Mueller and Sachs, 2015) can be the choice of tool for harnessing the microbiome for beneficial outcomes in agriculture and food industries. This technology proposed to manipulate the microbiome without culturing and move beyond current technologies such as the use of selective antibiotics and probiotics (Sheth *et al*., 2016). Synthetic biology will play an important role, to engineer novel but predictable functions in crop probiotics which upon addition to plant and soils will manipulate the microbiome and/or its activities in a predicted fashion. For example, bacteria could be engineered to modulate microbiomes or crop physiology by secreting specific chemicals, which in turn enhance crop resilience against resource and biotic stresses by stimulating the activities of beneficial microbiomes. When fully functional, these tools have the potential to revolutionize agricultural productivity and bring similar levels of productivity gains as observed during the green revolution. However, this is a mid‐ to long‐term goal for larger scale uses in agro‐ecosystem, given the complexities of the soil microbiota and the variety of signal molecules they utilize.

## Personalized food and nutrient security

In developing countries, the focus will be to increase agricultural productivity to ensure food security, whereas in developed countries, nutrient security and healthy food will become main policy drivers. An emerging concept is personalized diet/nutrients for better health outcomes. This will require food to be grown differently to minimize chemical contamination and reduce the concentration of natural allergens. Personalized diets will explicitly consider individual genetics, physiology and differences in microbiomes and their metabolic activities. Initial research supports the case for personalized diets as no two individuals respond identically to the same food, suggesting a key role for host–microbiome interactions in nutrient outcomes. For example, an important role in glucose haemostasis and obesity has been found in the gut microbiota. Together, this evidence challenges the traditional concept of a healthy diet with an optimized diet based on the unique host–microbiome make‐up (Zeevi *et al*., 2015). In future, people will be grouped based on their microbiomes for personalized diets. This can herald a new era of healthy lifestyle and prevention of metabolic (diabetes, heart disease) and physiological (allergy to natural compounds) conditions. In addition, probiotic cocktails will be developed and used to suppress known allergens or to affect nutritional uptake for specific food to minimize the negative impact of allergens on sensitive individuals.

## Current global initiatives

There has been tremendous interest in harnessing the microbiome for increasing agricultural productivity. In 2016, two key initiatives have been launched which explicitly recognize the potential of the microbiome approach. (i) The White House has launched the US microbiome initiative on 14 May 2016 with an investment of $450 million to enhance innovation and commercialization and for developing new, related industries. Crop and soil microbiomes are a core component of this initiative and are working closely with the Phytobiome initiative to ensure success. (ii) The EU Commission has launched the International Bioeconomy Forum (IBF) on 13 October 2016, and harnessing microbiomes for food and nutritional security is their first and key component, along with regional economic growth and job creation. Both initiatives envisage public–private partnership models as the key for rapid innovation and commercialization of products. There are also a number of large and small industries investing heavily in microbiome research, which is clear recognition of the commercial benefits of microbiome research with critical environmental and social benefits. For example, it is predicted that in the EU, a higher number of bio‐pesticides will be sold compared to chemical pesticides by 2020. The agricultural and nutrient sector is a key area of development in microbial biotechnology along with health sector and will be an important driver of global economic growth and social and environmental sustainability.

## Concluding remarks

In summary, we envisage the development of technologies which will allow the manipulation of the crop microbiome *in situ*. These technologies will become an integral part of the sustainable increase in agricultural productivity ensuring food and nutrient security for future global populations. If this is to be achieved, both theoretical and technological advancements are needed (Box 1), utilizing multidisciplinary approaches to integrate emerging technologies (omics, 3D printing, synthetic biology) with more traditional approaches of microbial ecology, plant eco‐physiology and genetics. These approaches will then be further embedded with remote sensing, satellite and sensor‐based technologies with the ability to handle big data to realize the true potential of microbiome tools in agriculture and food sectors. In addition, challenges associated with social and regulatory policies will require simultaneous attention. Public acceptance of microbiome‐based products will be crucial for the success of these technologies, and multidirectional communication among all stakeholders will ensure success. Standardization of regulatory requirements at intergovernmental levels will provide easy access to the market, but at the same time ensure efficacy and safety of these products to maintain public confidence in technologies. There are significant challenges to achieve the potential microbiome approach for food and nutrient security, but these are dwarfed by the potential economic, environmental and social benefits of taking this approach. For example, in addition to food and nutrient security, microbiome tools can substantially increase economic performance by commercializing new products, improve environmental health by reducing chemical contamination and create jobs in green industries.

Box 1 **Key steps towards successfui use of microbiome tools for food and nutrient security**

**Table:** 

Technical steps	Outcomes
1. Characterization of crop microbiome of different verities	Identification of core microbiomes
2. Relationship between phytomicrobiome and yield and quality	Role in nutrient acquisition and resilience against biotic and abiotic stresses
3. Identification signal of molecules (signalome) used by plant and microbes for two‐way communication	Mode of communication between crop and microbiomes
4. Development of sustainable biochemical and engineering technologies to promote activities of beneficial microbiome	Ability to match supply with crop demand for nutrient and protection
5. Validation/large‐scale production and commercialization	Sustainable increase of food production and quality to achieve food and nutrient security
